# Personal recovery in psychosis: neurobiological basis

**DOI:** 10.1192/j.eurpsy.2025.2172

**Published:** 2025-08-26

**Authors:** J. J. M. Jambrina, N. Á. Alvargonzález, L. P. Gómez, M. A. R. Cortina

**Affiliations:** 1 Psychiatry, Hospital Universitario San Agustín, Avilés, Spain

## Abstract

**Introduction:**

Personal recovery in psychosis is one of the fields where research and clinical activities are growing faster. But the concept lacks conceptual clarity and internal consistency. As a result, most of the studies evaluating its efectiveness may be biased. As in any other intervention, great improvement in clinical evolution of the users has been claimed. And so, neurobiological changes should be described.

**Objectives:**

This study finds to develop a systematic review of studies describing changes surrounding the recovery process from a neurobiological level.

**Methods:**

Keywords hev been selected in order to find all studies focussing on personal recovery in psychosis from its neurobiological basis. Qualitative studies of personal recovery will be excluded. Cross-sectional, longitudinal as well as intervention studies will be included.

**Results:**

The systematic review is underway. The study protocol will be registered at PROSPERO database.

**Conclusions:**

Most studies researching about personal recovery have been developed taking “personal recovery” as an outcome marker. But this is not enough. We need to clarify the neurobiological basis underlying this concept.

**Disclosure of Interest:**

None Declared

